# Supplementary material for: Competing social identities and intergroup discrimination: Evidence from a framed field experiment with high school students in Vietnam
Source: PLoS One. 2021 Dec 9;16(12):e0261275. doi: 10.1371/journal.pone.0261275 (PMC8659309; doi:10.1371/journal.pone.0261275)
Supplement: S1 Appendix — (PDF) [file pone.0261275.s001.pdf]

S1 Appendix. School Characteristics

Table A. French teaching curricula in Vietnam

	Hours per year	Number of years	Number of enrolled students	Location
French (major)	100 - 150	7	51,672	All over the country
French (intensive)	200 - 350	3	1605	All over the country
French (minor)	75	3	45,262	20 provinces
French bilingual classes	250	7-12	14,630	18 provinces

Data adapted from French Embassy in Vietnam – 2010.

Table B. General statistics of Ho Chi Minh City versus Vietnam

STATISTICS IN 2016	HO CHI MINH CITY	VIETNAM
Area (Km^2)	2,061Km^2	331,231Km^2
Average population	8,298 million	92,695 million
Population density where the students live (per km^2)	4,025	280
In-migration rate (2005 - 2016)	19.40%	4.60%
Out-migration rate (2005 - 2016)	7.70%	4.60%
Sex ratio of population in HCMC (Males per 100 females)	91.8	97.3
Salary per capita (per year)	$AUD 2,196	$AUD2,314

Data adapted from General Statistics Office of Vietnam – 2016)
