# Supplementary material for: Competing social identities and intergroup discrimination: Evidence from a framed field experiment with high school students in Vietnam
Source: PLoS One. 2021 Dec 9;16(12):e0261275. doi: 10.1371/journal.pone.0261275 (PMC8659309; doi:10.1371/journal.pone.0261275)
Supplement: S2 Appendix — (PDF) [file pone.0261275.s002.pdf]

S2 Appendix. Experimental Instructions

GENERAL EXPLANATIONS FOR PARTICIPANTS

As part of today's experiment, we will be performing some tasks. The funding for this research has been provided by the *Queensland University of Technology* and any money that you end up with will be yours to take home. You will be paid for one of the tasks. I will throw a dice at the end of the tasks in front of everyone to determine which task you will be paid for. In addition to any earnings, you might have in this task, you will be given **VND 50,000** as a participation fee.

We are about to begin the first task. Please listen carefully. It is important that you understand the rules of the task properly. If you do not understand, you will not be able to participate effectively. We will explain the task and go through some examples together. There is to be no talking or discussion of the task amongst you. There will be opportunities to ask questions to be sure that you understand how to perform each task. At any time whilst you are waiting during this experiment, please wait at your seat and do not do anything unless instructed by the experimenter. Also do not look at other's responses at any time during this experiment. If at any time you decide that you are not happy with the task you have been invited to perform, you can decide not to participate.

After we have completed all the tasks, I would like you to answer some questions about yourself. Please take your time and answer honestly and as accurately as possible. You will not be identified, and your survey answers will only be used for this experiment and will only be used by the researcher(s) involved in this project.

Finally, stapled behind this page is a slip of paper with your ID# on it. Please keep this page with the stapled ID# with you at all times. Do not show this ID# to anyone or allow it to be visible to anyone during or after this experiment. You will need to present this page with the stapled ID# to the cashier at the end of the experiment in order to receive your payment. If you are ready, then we will proceed. Please turn the page and follow along with the experimenter.

TASK #1 INSTRUCTIONS

This task is played by pairs of individuals. Each pair is made up of a Player 1 and a Player 2**.** Each of you will play this task with someone from this group. However, none of you will know exactly with whom you are playing. You will never find this out.

Each Player 1 has VND 60,000. No money will be given at this point. All actual payments will be made at the end of the experiment if this task is chosen as the one that you will be paid for.

Player 1 must decide how to divide this money between him or herself and Player 2. Player 1 must allocate between VND0 and the total VND 60,000 to Player 2. Player 2 takes home whatever Player 1 allocates to him/her, and Player 1 takes home whatever he or she does not allocate to Player 2.

Let’s go through an example:

Imagine that Player 1 chooses to allocate VND 30,000 to Player 2. Then, Player 2 will go home with VND 30,000 (VND 30,000 given). Player 1 will go home with VND 30,000 (VND 60,000 minus 30,000 equals 30,000).

Note that this is an example only. The actual decision is up to you.

Each of you will play as both Player 1 and Player 2 in this task. Each of you will be paired with two different individuals. The important thing to remember is that you will NOT be paired with the same person twice and you will always remain anonymous to each other. No-one will be told who they are paired with.

We will hand out all forms that you will record your decisions on throughout this experiment in envelopes to ensure this. If this task is chosen for payment, I will then toss a coin to determine which pairing you will be paid for. So, for any given toss of the coin, half of you will go home with what you kept as Player 1, half of you will go home with what the Player 1s have given you.

Are there any questions? If you are ready, we will proceed. You will convey your decision using the form provided. There is an example question and a table for you to record your decision. Please complete the example question first and then fill in Boxes A and B of the table. Once done, please place your form back into the envelope, raise your hand and we will collect the form from you.

Player ID #: ____________________

Paired Player ID # (For experimenter use only) : _______________

FORM FOR RECORDING DECISIONS FOR TASK #1

Before you fill out this form, please complete the example below:

1. Say you are Player 1 and you have 60,000. You choose to give 20,000 to Player 2. How much will you have and how much will Player 2 have? Player 1(yourself):_______________ Player 2:________________

2. When you have completed the example above, please enter the amount, in dollars, that you wish to keep and the amount that you wish to give to Player 2 in the table below.

	Total amount	60,000
A	Amount I wish to keep	
B	Amount I wish to send to anonymous Player 2	

When you have made your decision as Player 1, your task is complete. Please place your form back into the envelope, raise your hand and we will collect the form from you.

TASK #2 INSTRUCTIONS

We are about to begin the second task. Please listen carefully to the instructions. This task is performed by pairs of individuals. Each of you will play this task with someone from this group. Each pair is made up of a Player A and a Player B. Each Player A has VND 40,000. No money will be given at this point. All actual payments will be made at the end of the experiment if this task is chosen as the one that you will be paid for.

Each Player A will have the opportunity to keep all of 40,000 to himself/herself or allocate some or all of it to a Player B. However, each VND 1000 that Player A sends to Player B will be *tripled* by the experimenter and given to player B.

Player B will then have an opportunity to keep all of the money sent to him/her from Player A or to send some or all of it back to Player A. This time the money will *not* be tripled again. The experiment ends at this point.

Player B takes home whatever money that he/she does not give to Player A. Player A takes home whatever he/she did not give to Player B and whatever money Player B gave back.

Here are 2 examples of what could happen:

1) Say Player A gives Player B 10,000. This will be tripled, and it will be 30,000 when it reaches Player B. Then Player B sends back to Player A 10,000. Then Player A will have 40,000 (40,000 minus the 10,000 sent to Player B and plus the 10,000 sent back by Player B). Player B will have 20,000 (30,000 minus the 10,000 sent back to Player A).

2) Say Player A gives Player B 30,000. This will be tripled, and it will be 90,000 when it reaches Player B. Then Player B sends back to Player A 40,000. Then Player A will have 50,000 (40,000 minus the 30,000 sent to Player B and plus the 40,000 sent back by Player B). Player B will have 50,000 (90,000 minus the 40,000 sent back to Player A).

Note that these are only examples. The actual decisions are up to you. Each of you will play as both Player A and Player B in this task. Each of you will be paired with two different individuals. In one pair you will be Player A and in the other pair you will be Player B. So you will play this task once as Player A and once as Player B. The important thing to remember is that you will NOT be paired with the same person twice and you will always remain anonymous to each other. No-one will be told who they are paired with. If this task is chosen for payment, I will then toss a coin to determine which pairing you will be paid for. So for any given toss of the coin, half of you will go home with what you kept as Player A, half of you will go home with what the Player As have given you.

Are there any questions? If you are ready, we will proceed. You will convey your decisions using the form provided. Please turn over the page and look at the form that you will record your decision on. I will read through the form first. Please do not write anything until instructed to.

Player ID #: ____________________

Paired Player ID # (For experimenter use only): _______________

FORM FOR RECORDING DECISIONS FOR TASK #2

Part A

Before you fill out this form, please complete the example below:

1. You are Player A and you have 40,000. You choose to give 10,000 to Player B. How much will Player B have? VND______ x_______ =____________ Player B decides to send 15,000 back. How much will you have in total and how much will Player B have in total?

Player A: $_____ - $_____+$_____= _____ Player B: $_____-$_____ = _____

When you have completed the example above, please fill out Boxes A, B and C of the table below. When you have made your decision as Player A, your task as Player A is done at this point. Once you have completed Boxes A, B and C, raise your hand and I will collect the form from you. You will be informed of how much the Player B gave back to you at the end of the experiment when you collect your payment.

2.

**Your decision as Player A**		
	Starting amount	40,000
A	Amount I wish to keep as Player A	
B	Amount I wish to send to anonymous Player B	
C	Amount that Player B will receive (Box B x 3)	

When you have completed Part A, please read the instructions for Part B over page.

FORM FOR RECORDING DECISIONS FOR TASK #2

Part B

Recall that you will also be a Player B in another pairing. I will record how much Player A in this pairing has sent to you in Box D when I collect your forms after you fill in Boxes A, B and C. The amount in Box D will already be tripled. I will then return the form to you and you will then decide how much money to keep and how much to send back to Player A. You will need to fill in Boxes E and F.

When you have read the above paragraph, place your form for Task #2 into the envelope, raise your hand and I will collect your form from you.

3.

**Your decision as Player B**		
D	Amount received from Player A *(already tripled)*	[pre-filled by the experimenter]
E	Amount I wish to keep	
F	Amount I wish to send back to Player A	

Once you have completed boxes E and F, your task is done. Please place this form into your envelope, raise your hand, and I will collect the form from you.

TASK #3 INSTRUCTIONS

We are about to begin the third task. Please listen carefully to the instructions.

In this task, you will be given 20,000. No money will be given at this point. All actual payments will be made at the end of the experiment if this task is chosen as the one that you will be paid for. You have the opportunity to invest a portion of this amount (between 0 and 20,000).

The investment: There is an equal chance that the investment will fail or succeed. If the investment fails, you lose the amount you invested. If the investment succeeds, you receive 3 times the amount invested.

How do we determine outcome of investment: After you have chosen how much you wish to invest, you will toss a coin to determine whether you win or lose if this task is chosen for payment. If the coin comes up heads, you win three times the amount you chose to invest. If it comes up tails, you lose the amount invested. You will toss the coin when you come to collect your payment at the end of the experiment.

Here are some examples:

1. You choose to invest nothing. You will get 20,000 for sure if this task is chosen for payment.

2. You choose to invest all of the 20,000. Then if the coin comes up heads, you get 60,000. If the coin comes up tails, you get 0.

3. You choose to invest 10,000. Then if the coin comes up heads, you get 40,000 (10,000 x 3 + 10,000 = 40,000). If the coin comes up tails, you get 10,000.

Do you have any questions? If you are ready, we will proceed. Please fill in the amount that you would like to invest in the form on the other side of the page and raise your hand when you are done. I will then collect the forms.

Player ID #: ___________________________

FORM FOR RECORDING DECISION FOR TASK #3

Amount I wish to invest	

TASK #4 INSTRUCTIONS

We are about to begin the fourth task. Please listen carefully to the instructions. All the money that you earn from this task is yours to keep and will be given to you at the end of this experiment if this task is chosen as the one that you will be paid for.

For Task 4, you will be asked to calculate the sum of five randomly chosen two-digit numbers. <u>*For example:*</u>

22	17	83	61	49	*Answer here*

You will be given 5 minutes to do as many sums as possible and you must complete the sums in order. You cannot use a calculator to calculate the sums, however you are welcome to make use of the provided scratch paper. Your answers to the problems are anonymous. You can choose one of two payment options for this task.

Option 1:

If you choose this option, you get 5,000 for each problem that you solve correctly in the 5 minutes. Your payment does not decrease if you provide an incorrect answer to a problem.

Option 2:

If you choose this option, you will be randomly paired with someone and your payment depends on your performance relative to that of the person that you are paired with. If you solve more problems correctly than the person you are paired with, you will receive 10,000 per correct answer. If you both solve the same number of problems, you will receive 5,000 per correct answer. If you solve less than the person you are paired with, you will receive $0.

Note that what you will earn does not depend on the decision of the person that you are paired with; it only depends on your own choice of payment, your performance and their performance. Here are some examples of what could happen:

1. You choose option 1. You complete 10 sums correctly. You will receive 10 x 5,000 = 50,000.

2. You choose option 2. You complete 8 sums correctly. The person that you are paired with completes 7 sums correctly. You will receive 8 x 10,000 = 80,000.

Note that these are examples only. The actual decision is up to you.

Are there any questions before we begin? If you are ready, we will proceed. Before we begin the task, please fill out the questions on the form over page and raise your hand when you are done.

Player ID #: ____________________

Paired Player ID # (For experimenter use only): _______________

QUESTIONS FOR TASK #4

Please answer the following questions:

1. Suppose you choose Option 1. You complete 11 sums correctly at the end of 5 minutes. How much money do you receive? _______ x VND________ = ________________

2. Suppose you choose Option 2. You complete 7 sums correctly. The person you are paired with completes 6 sums correctly. How much money do you receive? _______ x VND________ = ________________

3. How many sums do you think you can complete correctly in 5 minutes? _____________________

4. If we were to rank everyone's performance in the group from best to worst, where do you think you would fall compared to the average person? *Please place a tick next to the rank that you think applies to you.* __ (very above average) __ (above average) __ (average) __ (below average) __ (very below average)

5. We now ask you to choose how you want to be paid: according to option 1 or option 2? _____________________

6. What was your decision based on?

7. If you chose Option 1, did your decision depend on the payment rate under Option 2? If so, what payment rate would have convinced you to choose Option 2?

Please place the form back into the envelope when you are done, raise your hand and I will collect the form from you. When I have collected everyone's forms, I will then hand out the sums sheets. Please do not talk while you are waiting and do not turn over the sheets. Raise your hand if you have any questions.

Player ID #: __________________

Paired Player ID # (For experimenter use only): _______________

FORM FOR RECORDING ANSWERS FOR TASK #4

Add up the numbers in each row and record your answer in the blank box at the end

40	39	57	82	26	
82	18	59	79	35	
38	52	41	47	22	
78	10	59	81	78	
18	58	40	49	53	
42	61	53	49	41	
64	98	27	38	78	
86	25	75	50	64	
90	58	98	89	45	
72	19	59	40	37	
45	41	47	36	54	
33	68	68	55	67	
34	90	30	84	35	
13	67	20	45	44	
79	92	11	87	16	
13	21	96	53	65	
11	69	26	45	13	
13	37	54	94	41	
45	14	17	50	67	
84	13	98	66	21	
74	83	64	52	91	
17	93	55	69	89	
87	80	32	52	28	
58	61	96	58	21	

95	56	47	10	30	
98	24	32	83	53	
87	24	78	75	61	
11	30	84	19	12	
29	91	52	70	88	
19	32	26	46	10	
48	44	87	21	20	
81	71	47	84	33	
80	34	83	96	22	
33	15	24	33	22	
54	41	79	18	89	
46	71	87	39	12	
34	18	44	72	57	
95	86	52	24	32	
72	86	34	12	77	
11	69	26	45	13	

TASK #1 INSTRUCTIONS*

This task is played by bilingual students ONLY and performed by pairs of individuals. Each pair is made up of a Player 1 and a Player 2**.** Each of you will play this task with *a bilingual student from Minh Khai High School*. However, none of you will know exactly with whom you are playing. You will never find this out.

Each Player 1 has VND 60,000. No money will be given at this point. All actual payments will be made at the end of the experiment if this task is chosen as the one that you will be paid for.

Player 1 must decide how to divide this money between him or herself and Player 2. Player 1 must allocate between VND0 and the total VND 60,000 to Player 2. Player 2 takes home whatever Player 1 allocates to him/her, and Player 1 takes home whatever he or she does not allocate to Player 2.

Let's go through an example:

Imagine that Player 1 chooses to allocate VND 30,000 to Player 2. Then, Player 2 will go home with VND 30,000 (VND 30,000 given). Player 1 will go home with VND 30,000 (VND 60,000 minus 30,000 equals 30,000).

Note that this is an example only. The actual decision is up to you.

Each of you will **only be played as Player 1** in this task. We will hand out all forms that you will record your decisions on throughout this experiment in envelopes to ensure this. If this task is chosen for payment, you will go home with what you kept as Player 1.

Are there any questions? If you are ready, we will proceed. You will convey your decision using the form provided. Please turn over the page and look at the form that you will record your decision on. There is an example question and a table for you to record your decision. Please complete the example question first and then fill in Boxes A and B of the table. Once done, please place your form back into the envelope, raise your hand and we will collect the form from you.

TASK #2 INSTRUCTIONS*

We are about to begin the second task. Please listen carefully to the instructions. **This task is also played by bilingual students ONLY** and performed by pairs of individuals. Each of you will play this task with *a bilingual student from Minh Khai High School*. However, you will always remain anonymous to each other. No-one will be told who they are paired with.

Each pair is made up of a Player A and a Player B. Each Player A has VND 40,000. No money will be given at this point. All actual payments will be made at the end of the experiment if this task is chosen as the one that you will be paid for.

Each Player A will have the opportunity to keep all of 40,000 to himself/herself or allocate some or all of it to a Player B. However, each VND 1000 that Player A sends to Player B will be *tripled* by the experimenter and given to player B.

Player B will then have an opportunity to keep all of the money sent to him/her from Player A or to send some or all of it back to Player A. This time the money will *not* be tripled again. The experiment ends at this point.

Player B takes home whatever money that he/she does not give to Player A. Player A takes home whatever he/she did not give to Player B and whatever money Player B gave back.

Here are 2 examples of what could happen:

1) Say Player A gives Player B 10,000. This will be tripled, and it will be 30,000 when it reaches Player B. Then Player B sends back to Player A 10,000. Then Player A will have 40,000 (40,000 minus the 10,000 sent to Player B and plus the 10,000 sent back by Player B). Player B will have 20,000 (30,000 minus the 10,000 sent back to Player A).

2) Say Player A gives Player B 30,000. This will be tripled, and it will be 90,000 when it reaches Player B. Then Player B sends back to Player A 40,000. Then Player A will have 50,000 (40,000 minus the 30,000 sent to Player B and plus the 40,000 sent back by Player B). Player B will have 50,000 (90,000 minus the 40,000 sent back to Player A).

Note that these are only examples. The actual decisions are up to you. Each of you will **only be played as Player A** in this task. If this task is chosen for payment, you will go home with what you kept as Player A and whatever amount sent back by Player B.

Are there any questions? If you are ready, we will proceed. You will convey your decisions using the form provided. Please turn over the page and look at the form that you will record your decision on. I will read through the form first. Please do not write anything until instructed to.
