# Supplementary material for: Competing social identities and intergroup discrimination: Evidence from a framed field experiment with high school students in Vietnam
Source: PLoS One. 2021 Dec 9;16(12):e0261275. doi: 10.1371/journal.pone.0261275 (PMC8659309; doi:10.1371/journal.pone.0261275)
Supplement: S3 Appendix — (PDF) [file pone.0261275.s003.pdf]

S3 Appendix. Additional Results

Table A. Mean comparison *t*-test and Wilcoxon rank-sum test exploring amount allocated between groups in the Dictator Game

Group 1		Group 2	Mean diff.	t-stat.	p-value	Z-stat.	p-value
**NC**-NC	vs.	**BC**-BC	-3.09	-1.97	0.499	-0.92	1
**NC**-BC	vs.	**BC**-NC	2.71	1.71	0.874	1.64	-0.5016
**BC**-NC	vs.	**BC**-BC	-21.07	-13.31***	< 0.001	-10.77***	< 0.001
**BC**-NC	vs.	**NC**-NC	-17.98	-11.44***	< 0.001	-9.93***	< 0.001
**NC**-BC	vs.	**BC**-BC	-18.36	-11.59***	< 0.001	-9.13***	< 0.001
**NC**-BC	vs.	**NC**-NC	-15.26	-9.71***	< 0.001	-8.27***	< 0.001
**BC**-BK	vs.	**BC**-BC	-11.92	-7.44***	< 0.001	-4.41***	< 0.001
**BC**-BK	vs.	**BC**-NC	9.15	5.72***	< 0.001	6.24***	< 0.001
**BC**-BK	vs.	**NC**-BC	6.44	4.02**	0.001	4.62***	< 0.001
**BC**-BK	vs.	**NC**-NC	-8.82	-5.55***	< 0.001	-3.53**	0.0021

Mean difference = Group 1 minus Group 2. The symbols ** and *** represent statistical significance at the 1%, and 0.1% levels, respectively. Both *p*-values are adjusted for multiple comparison using the Bonferroni method.

Table B. Mean comparison *t*-test and Wilcoxon rank-sum test exploring difference in amount sent between sessions in the Trust Game

Group 1		Group 2	Mean diff.	t-stat.	p-value	Z-stat.	p-value
**NC**-NC	vs.	**BC**-BC	-0.44	-0.3	1	-0.48	1
**NC**-BC	vs.	**BC**-NC	0.29	0.19	1	0.32	1
**BC**-NC	vs.	**BC**-BC	-26.07	-17.31***	< 0.001	-10.95***	< 0.001
**BC**-NC	vs.	**NC**-NC	-25.63	-17.13***	< 0.001	-10.54***	< 0.001
**NC**-BC	vs.	**BC**-BC	-25.79	-17.12***	< 0.001	-10.63***	< 0.001
**NC**-BC	vs.	**NC**-NC	-25.34	-16.94***	< 0.001	-10.22***	< 0.001
**BC**-BK	vs.	**BC**-BC	-22.01	-14.45***	< 0.001	-7.47***	< 0.001
**BC**-BK	vs.	**BC**-NC	4.06	2.67†	0.08	3.36**	0.0038
**BC**-BK	vs.	**NC**-BC	3.78	2.48	0.137	3.05*	0.0114
**BC**-BK	vs.	**NC**-NC	-21.57	-14.26	< 0.001	-7.04***	< 0.001

Mean difference = Group 1 minus Group 2. The symbols †, *, ** and *** represent statistical significance at the 10, 5%, 1%, and 0.1% levels, respectively. Both *p*-values are adjusted for multiple comparison using the Bonferroni method.

Table C. Mean comparison *t*-test and Wilcoxon rank-sum test exploring difference in amount returned between sessions in the Trust Game

Group 1		Group 2	Mean diff.	t-stat.	p-value	Z-stat.	p-value
NC-**NC**	vs.	BC-**BC**	5.03	1.92	0.556	0.88	1
NC-**BC**	vs.	BC-**NC**	-0.07	-0.03	1	-0.09	1
NC-**BC**	vs.	BC-**BC**	-33.93	-12.87***	< 0.001	-10.22***	< 0.001
NC-**BC**	vs.	NC-**NC**	-38.96	-14.88***	< 0.001	-11.17***	< 0.001
BC-**NC**	vs.	BC-**BC**	-33.86	-12.84***	< 0.001	-10.13***	< 0.001
BC-**NC**	vs.	NC-**NC**	-38.89	-14.85***	< 0.001	-11.07***	< 0.001
BC-**BK**	vs.	BC-**BC**	-28.26	-10.6***	< 0.001	-5.99***	< 0.001
BC-**BK**	vs.	NC-**BC**	5.67	2.13	0.341	4.12***	< 0.001
BC-**BK**	vs.	BC-**NC**	5.60	2.1	0.364	4.03***	< 0.001
BC-**BK**	vs.	NC-**NC**	-33.29	-12.57***	< 0.001	-6.89***	< 0.001

The symbol *** represents statistical significance at the 0.1% level. Both *p*-values are adjusted for multiple comparison using the Bonferroni method.
